# Effect of Chlorhexidine-containing Etch-and-Rinse Adhesives on Dentin Microtensile Bond Strength after Biological Loading

**DOI:** 10.3290/j.jad.b3801065

**Published:** 2023-01-12

**Authors:** Christina Boutsiouki, Roland Frankenberger, Susanne Lücker, Norbert Krämer

**Affiliations:** a Scientific Associate, Department of Pediatric Dentistry, Dental School, University of Marburg and University Medical Center Giessen and Marburg, Campus Giessen, Giessen, Germany. Performed the experiments, wrote the manuscript.; b Professor, Department of Operative Dentistry, Endodontics, and Pediatric Dentistry, Dental School, University of Marburg and University Medical Center Giessen and Marburg, Campus Marburg, Marburg, Germany. Supervision, data curation, proofreading.; c Research Associate, Department of Operative Dentistry, Endodontics, and Pediatric Dentistry, Dental School, University of Marburg and University Medical Center Giessen and Marburg, Campus Marburg, Marburg, Germany. Organization, supervision of experiments.; d Professor and Head, Department of Pediatric Dentistry, Dental School, University of Marburg and University Medical Center Giessen and Marburg, Campus Giessen, Giessen, Germany. Idea, study design, data analysis.

**Keywords:** biofilm, biological loading, bond durability, cariology, chlorhexidine gluconate, dentin bonding, microtensile bond strength.

## Abstract

**Purpose:**

This study compared a 2%-CHX dentin pre-treatment with three CHX adhesives (experimentally admixed 0.1% CHX in primer or bonding agent, or industrially added 0.2% CHX in universal adhesive) by evaluating dentin bond strengths after biological loading in a fully automated artificial mouth model.

**Materials and Methods:**

The occlusal dentin of 50 freshly extracted human third molars was exposed, and the teeth were randomly assigned to 5 groups according to the adhesive protocol (n = 10): 1. control, Scotchbond Multipurpose (3M Oral Care; CTRL); 2. 2% CHX dentin pre-treatment (DENT); 3. 0.1% CHX experimentally admixed into the primer (PRIM); 4. 0.1% CHX experimentally admixed into the bonding agent (BOND); 5. Peak Universal Bond containing 0.2% CHX (Ultradent; PEAK). The teeth were restored with composite resin. Microtensile bond strength testing (bonding area 0.46 mm^2^ ± 0.04 mm^2^, crosshead speed 1 mm/min) was performed after 24-h storage in distilled water (baseline) or after 2-day biological loading with *S. mutans* (demineralization 1 h / remineralization 5 h). The mode of fracture was recorded and exemplary sticks were evaluated under SEM.

**Results:**

CTRL exhibited significantly higher μTBS at baseline in comparison to PRIM (p = 0.000), BOND (p = 0.002), and PEAK (p = 0.000). After undergoing the caries model, CTRL demonstrated significantly lower μTBS compared to DENT (p = 0.000), PRIM (p = 0.008), and PEAK (p = 0.000). The same behavior was observed for BOND vs DENT (p = 0.000), PRIM (p = 0.003), and PEAK (p = 0.001). After biological loading, DENT (p = 0.041), PRIM (p = 0.000), and BOND (p = 0.000) exhibited significantly fewer adhesive fractures than CTRL.

**Conclusions:**

CHX addition to the primer protects dentin bond strength from declining after biological loading. Thus, it may offer some clinical advantage in terms of secondary caries inhibition around composite restorations. However, since loss of adhesion at baseline was less when 2% CHX was used as a dentin pre-treatment, it can be suggested as a safer option. so that bonding is not undermined by potential chemical interactions from CHX with the adhesives.

Oral cavity characteristics and components of the dentin still challenge the long-term stability of adhesive bonds and worsen the prognosis of direct restorations, despite the fact that contemporary adhesives present satisfactory immediate bonding performance. Degradation of the adhesive interface is the collective result of water, bacteria, and endogenous proteolytic enzymes, either matrix metalloproteinases (MMPs) or cysteine cathepsins (CCs), which are activated either iatrogenically due to low pH during adhesive procedures^33,48,53^ or during caries progression because of lactic acid production.^52^ Bacteria from the oral cavity can further threaten the restorations by adhering on restorative materials or more often in interfaces,^16^ causing secondary caries.^19,27,29^ Since hydrolysis is difficult if not impossible to prevent in a tissue which consists of ~ 22% water and 33% organic compounds, research has focused on ways to reduce the intrinsic enzymatic activity, and on antibacterial strategies to reduce the risk of secondary caries extrinsically.^4,17,21,40^

Chlorhexidine (CHX) is a cationic-bisguanide and possesses both bacteriostatic and bactericidal effects against Gram+ and Gram- species. Its antibacterial action against *S. mutans*^5^ was known long before the importance of CHX in restorative dentistry was recognized. CHX was first used as a dentin disinfectant and re-wetting agent prior to adhesive bonding,^22,41^ before realizing its activity against collagenases and gelatinases (MMPs) and more recently against CCs – specifically against CC-B, -K and –L.^33,40,48,53^ Because of its substantivity,^12^ CHX was still detectable in the hybrid layer after 5^33^ or even 10 years;^10^ however, the exact duration of its effect is not known. CHX can be delivered through dentin pre-treatment^17,34,40^ or admixed into the adhesives,^2,11,18,36,37,44^ but this raises some issues regarding its potential interference with the mechanical properties and bonding efficiency of the adhesives used as carriers.^25,38^ Inhibition of bacterial action via incorporation of antibacterial substances in restorative materials, cements, or adhesives has been extensively discussed.^15,16,26,43^ A review by the Cochrane Collaboration concludes that there is not enough clinical data to assess the ability of antibacterial restorative materials to prevent dental caries.^43^ Pre-treatment with CHX has been shown to suppress collagenolytic activities in dentin both in-vitro^17,21,40^ and in-vivo^8,9,13,20,23,45^ even at low concentrations.^21^ Only one adhesive with industrially incorporated 0.2% CHX is commercially available.^2,47^ The literature shows positive results regarding bond strength maintenance when CHX is experimentally mixed into the adhesive.^34,36,37,50,51,54,55^ According some studies,^11,37^ the degree of conversion of adhesives was not influenced when CHX was added, but they may become stiffer, as their elasticity decreased due to decreased E-modulus up to 48% compared to the controls.^11^ Solubility and water sorption of CHX adhesives remain unaffected after 28 days^51^ or longer.^18^ The inhibitory effect of CHX on MMPs seems to be dependant on the CHX contentration and application duration. According to Zhou et al,^57^ 0.5%-2% CHX incorporated into the primer of a two-step self-etching adhesive showed an anti-MMP effect at every concentration tested when applied for 20 s, but was only effective at the 2% concentration when dentin was treated for 2 min. In a study by Pomacóndor-Hernández et al^44^ monitoring bond strength of a self-etching CHX adhesive, 2% CHX solution was not merely added to the adhesive; it replaced liquid A of a two-bottle self-etch adhesive, which did not alter its bonding efficacy even after 6 months. No published data is available on CHX addition to etch-and-rinse adhesives. A single available in-vivo study with self-etch CHX adhesives reported no difference in retention rates of 126 restorations after 2 years.^3^ Although bacteria can negatively affect μTBS,^35^ Borges et al^4^ demonstrated the opposite, ie, no reduction of CHX adhesive bond strengths after a 4-h/day cariogenic challenge. To date, no clinical study is available which assesses the effect of biological loading on CHX adhesives and the ability of CHX to arrest bond degradation via its antibacterial action or because of suppression of collagenolytic enzymes activated during caries.^52^

Due to ethical problems with clinical studies using experimental adhesives containing CHX, in-vitro models have been developed to generate fundamental aspects of the carious process by simulating the oral microcosm chemically or bacterially. Caries models with bacteria provide a realistic simulation of bacterial loading,^1,6,30,31^ isolate potential influencing factors, and study larger samples without the ethical issues inherent in animal trials. Fundamental requirements for an effective caries model are: pH control, pH cycling to reproduce de- and remineralization phases, simulation of intraoral sugar effects for the demineralization phase, adjustment of the saliva effect and of sugar clearance for the remineralization phase, and choice of nutritient medium being exactly adjusted to the bacteria under investigation.^1,6, 30,31^

This study compared 2%-CHX dentin pre-treatment with three CHX adhesives (experimentally admixed 0.1% CHX in the primer or bonding agent, or industrially added 0.2% CHX in a 2-step adhesive) by evaluating dentin bond strengths after biological loading with *S. mutans* in a fully automated artificial mouth model. The null hypothesis was that CHX adhesives do not show different bond strengths after biological loading.

## Materials and Methods

Fifty freshly extracted human third molars were collected with the informed consent of the patients and upon approval of the local ethics committee (AZ 143/09). The teeth were cleaned, examined with 3X magnification loupes for caries, fractures, or defects, and stored in 0.5% chloramin-T solution (chloramin-T trihydrate, Carl Roth; Karlsruhe, Germany) at 5°C-7°C for up to 30 days. Roots were removed with a slow-speed diamond saw (Isomet 1000, Buehler; Lake Buff, IL, USA), and occlusal dentin was exposed in a grinding machine (Beta Grinder-Polisher, Buehler) with silicon carbide sandpaper of roughness P 600 – Grit 360, followed by decreasing roughness P 1200 – Grit 600 (Silicon Carbide Grinding Paper Grit 360 and Grit 600, Buehler Met II, Buehler), under water irrigation. Dentin surfaces were dried and checked for enamel remnants using 3X-magnification loupes with light. Subsequently, dentin was further manually polished with P 1200 – Grit 600 sandpaper for 60 s, performing “figure-8” routes, in order to remove debris and create a standardized, even smear layer.^32,39^

Teeth were randomly divided into 5 groups (n = 10) according to the adhesive protocol used (Table 1). The experimental adhesives were composed on the basis of a 3-step adhesive (Adper Scotchbond MP, 3M Oral Care; St Paul, MN, USA).^55,56^ Two percent chlorhexidine digluconate (Gluco-Hex 2% Solution, Cerkamed; Stalowa Wola, Poland) was used. A commercially available universal adhesive containing 0.2% CHX was also used in etch-and-rinse mode (Peak Universal Bond with 0.2% chlorhexidine, Ultradent). For the experimental adhesives, CHX was admixed into the primer or bonding agent of Adper Scotchbond MP (3M Oral Care) (Table 2), reaching a final CHX concentration of 0.1%. Adper Scotchbond Primer and CHX/Adper Scotchbond Bonding Agent and CHX were thoroughly mixed with a 2-mm brush applicator for 20 s and the mixture was allowed to set for 10 s before application of the experimental CHX adhesive on the tooth. To standardize the procedure, a fresh batch of CHX primer/CHX adhesive was prepared for each tooth. The following groups were formed: 1. control (CTRL); 2. 2% CHX dentin pre-treatment (DENT); 3. 0.1% CHX experimentally admixed into the primer (PRIM); 4. 0.1% CHX experimentally admixed into the bonding agent (BOND); 5. Peak Universal Bond containing 0.2% CHX (Ultradent; PEAK). Groups PRIM and BOND are experimental CHX adhesives, and CHX in group PEAK is industrially added. The application procedures of the adhesives is presented in Table 2. Composite buildup followed. The first layer of composite resin (Filtek Z250, 3M Oral Care) was applied and thinned to 0.5 mm (Comporoller 5300, Kerr Dental; Orange, CA, USA). Consecutive composite layers of 1 mm thickness were placed, up to approximately 6 mm. Each layer was separately polymerized for 40 s with an LED polymerization unit at 1200 mW/cm^2^ light intensity (Elipar, 3M Oral Care). Specimens were stored in distilled water at 37°C (Incubator IP20 Function Line, Heraeus Kulzer; Hanau, Germany) for 24 h. Teeth were then mounted on a microtome table with wax (Supradent-Wachs, Pluradent; Frankfurt am Main, Germany), such that the composite buildup faced downwards and, using a precision microtome (Isomet 5000 Linear Precision Saw, Buehler), cuts were made horizontally and vertically to form sticks. Rectangular (0.68 mm x 0.68 mm) sticks with 0.46 mm^2^ (±0.04 mm^2^) bonding area were produced. Approximately 18-25 sticks were fabricated from each tooth.

**Table 1:** Materials used according to manufacturer’s information

Product, manufacturer	Type	Composition (% by wt)	Lot
Adper Scotchbond Multipurpose Adhesive, 3M Oral Care; St Paul, MN, USA	3-step etch-and-rinse	Scotchbond Etchant: 55-65% water, 30-40% phosphoric acid, 5-10% synthetic amorphous silica Primer: 40-50% water, 35-45% 2-hema, 10-20% copolymer of acrylic and itaconic acids Bonding: 60-70% bis-GMA, 30-40% 2-HEMA, <0.5% triphenylantimony	516827 N510460 N515442
Peak Universal Bond with 0.2% chlorhexidine, Ultradent; South Jordan, UT, USA	Universal adhesive used in etch-and-rinse mode	Ultra-etch:<45% phosphoric acid Adhesive: <20% ethyl alcohol, ≤16% 2-hema, ≤6% methacrylic acid, <0.3% chlorhexidine di(acetate), 7.5% fillers	B8ZG1
Gluco-CHeX 2%, Cerkamed; Stalowa Wola, Poland	Chlorhexidine digluconate	2% chlorhexidine gluconate	1806131
Filtek Z250, 3M Oral Care	Composite resin	75-85% silane treated ceramic, 1-10% bis-EMA, 1-10% UDMA, 1-10% bis-GMA, <5% TEG-DMA, <5% aluminum oxide, <0.5% benzotriazol, <0.2% EDMAB	N512895 N561790 N608865 N635023

**Table 2:** Application directions of the adhesives used

Bonding system	Group	Preparation of CHX adhesives	Application steps
Adper Scotchbond Multipurpose Adhesive, 3M Oral Care	CTRL	-	1, 2, 3, 4, 5, 6, 7
	DENT	-	1, 2, 9, 3, 4, 5, 6, 7
	PRIM	Mix 0.5 μl of 2% CHX digluconate and 9.5 μl Scotchbond Primer	1, 2, 3 (CHX PRIMER), 4, 5, 6, 7
	BOND	Mix 0.5 μL of 2% CHX digluconate and 9.5 μl Scotchbond Bonding	1, 2, 3, 4, 5 (CHX BOND), 6, 7
Peak Universal Bond with 0.2% chlorhexidine, Ultradent	PEAK	CHX industrially admixed	1, 2, 8, 4, 7

1. Etch enamel (30 s) and dentin (15 s) with phosphoric acid; 2. rinse for 30 s and dry; 3. apply primer with an applicator brush to enamel and dentin for 10 s; 4. air dry gently for 5 s from 10 cm distance; 5. apply bonding with an applicator brush to enamel and dentin for 10 s; 6. air-thinning; 7. light cure for 20 s; 8. apply adhesive with applicator sponge and scrub for 10 s; 9. apply 2% CHX on dentin with an applicator sponge for 10 s and air dry.

**Fig 1:**
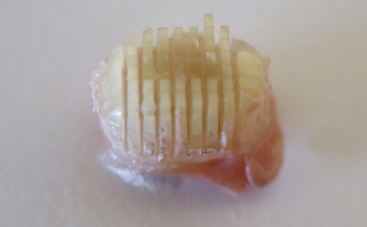
Composite-dentin sticks attached to the underlying composite resin surface. Sticky-wax is placed under the composite resin, so that specimens can be mounted on a chewing simulator plate.

Sticks which proceeded directly to microtensile bond strength (μTBS) testing were considered as baseline. Sticks planned for biological loading in the caries model remained attached to the underlying composite (Fig 1) and were mounted with sticky-wax (Supradent-Wachs, Pluradent) to chewing simulator plates (custom-made plates, Festo Systemtechnik; Denkendorf, Germany). The sticks were disinfected for 60 min in a disinfectant solution (Braunol 7.5 g povidone-iodine, B Braun; Melsungen, Germany). The solution was carefully stirred every 10 min. No ethanol was used in order to protect the already sectioned sticks and dentin from dehydration.

**Fig 2:**
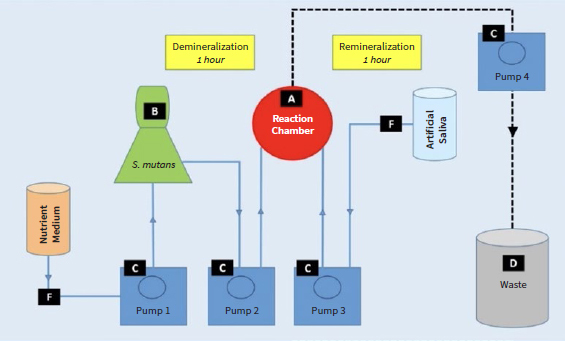
Schematic drawing of caries model set-up. A: reaction chamber for specimens; B: reservoir-container with *S. mutans*; C: pumps which enable movement of the media; D: waste container; F: input lines for artificial saliva and nutrient medium.

Subsequently, specimens to be used in the caries model were transferred into the sterilized reaction chamber (300-4100 Reusable Filter Holder with Receiver, Thermo Fisher Scientific Nalgene Labware; Rochester, NY, USA) in a Clean Bench (Clean bench, Thermo Fisher Scientific). Biological loading with *S. mutans* (DSMZ 20523) followed for two 2 days in a fully automated caries model (Fig 2), with consecutive demineralization (1 h) and remineralization phases (5 h).^1,6,31,46,49^ A total of 8 demineralization phases were planned. Subsequently, they were cultured in nutrient medium (Schaedler Broth, Becton Dickinson; Franklin Lakes, NJ, USA) for 12 h at 37°C, diluted to 1:10, incubated for 9 h, followed by inoculating the bacteria in the caries model. The average microbial load at the end of each cycle was 10^6^ microbes/ml. Each demineralization was induced after incubating the bacterial solution for 6 h in the same medium (Schaedler Broth, Becton Dickinson). Remineralization was achieved with artificial saliva^1,6,31,46,49^ having a pH=7 and containing phosphate buffer (2.2 mmol/l KH_2_PO_4_, 4.59 mmol/l KH_2_PO_4_). During each remineralization phase, the reaction chamber was rinsed four times with artificial saliva; in the final rinse process, the artificial saliva remained in the chamber. Temperature (37°C) and pH (pH=7 during remineralization and ca. 4.3 during demineralization) were monitored throughout the experiment (pH Electrode Blueline, SI Analytics; Mainz, Germany). Purity control and estimation of average microbial load was performed before inoculation and after the end of the experiment, in order to exclude the possibility of external contamination. During purity control, the bacterial solution was diluted up to 10^-5^ to enable colony counting on the cultured plates. Thereafter, specimens underwent μTBS testing.

μTBS testing was performed using the Universal Tensile and Pressure Testing System TC 550 (SyndiCAD; Munich, Germany) with its accompanying operating software (TC-550 Zug-/Druck-Messsoftware V3_1, Munich, Germany). Each stick was attached to two metal plates with flowable composite resin (Dyract Flow, Dentsply Sirona; Konstanz, Germany). In order to ensure horizontal placement and even tensile-force distribution, avoiding simultaneous shear strain, the metal plates were of equal thickness. Sticks were loaded at a speed of 1 mm/min until fracture. The type of bond failure (adhesive, cohesive in composite, cohesive in dentin, mixed) was assessed under light and 4X magnification from a Magnifier Lamp (1.75/4X, Model No: 8093, bulb: 12W, MBFZ toolcraft; Spalt, Germany) by a single examiner. Representative specimens were prepared for SEM examination, as follows. Organic content was removed by immersing sticks in 4% NaOCl solution (NaOCl, Carl Roth; Karlsruhe, Germany) for 20 min, rinsing with distilled water, then placement in 20% HCl (HCl, Sigma-Aldrich; St Louis, MO, USA) for 30 min, followed by a second rinse with distilled water, and finally immersed in 37% HCl for 6 h until the dentin was completely dissolved.^42^ Sticks were then dehydrated by immersion in an ascending (60%-100%) ethanol series and finally immersed in 1,1,1,3,3,3-hexamethyldisilazane (Merck Schuchardt; Hohenbrunn, Germany) before gold-sputtering.^42^ Representative areas of the adhesive interface (composite-dentin) of the selected sticks were investigated under SEM (SEM Amray Model 1610 Turbo, Amray; Bedford, MA, USA) at 1000X magnification. The hybrid layer and quality of resin tags were observed and compared with the control group.

Sticks were excluded from the study when the following criteria were met: i) inadequate length of composite and dentin (>3 mm); ii) voids in the adhesive or in composit; iii) signs of dentin caries or any other macroscopically visible flaw; iv) incorrect dimensions of the adhesive area; v) non-rectangular adhesive surface; vi) failure during separation from the underlying composite. Bond failure during sectioning was evaluated as a pre-test failure. All pre-test failures were assigned a bond strength of zero.

Statistical analysis was performed with SPSS v 15.0 (SPSS; Chicago, IL, USA) for Windows. The level of significance was set at 0.05. Normal distribution of the μTBSs obtained was checked with the Kolomogorov-Smirnov test. Two-way ANOVA was performed to evaluate the interaction between the factors “biological loading” and “CHX adhesive”. Homogeneity of variances was checked with Levene’s test. Significant differences between μTBSs were investigated using the Bonferonni test; Pearson’s chi-squared test was applied to differences between failure modes of the tested adhesives.

## Results

**Fig 3:**
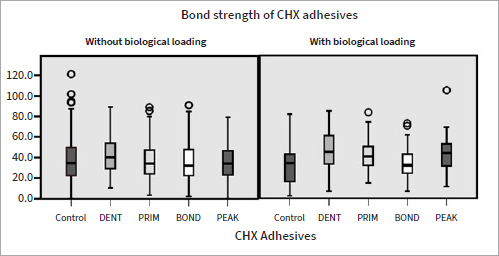
Boxplot with μTBS in MPa at baseline (without biological loading) and after biological loading in the caries model.

Microtensile bond-strength means, standard deviations (Table 3), and medians (Fig 3) of the adhesives at baseline and after biological loading in the caries model are reported. Data were compared within the groups (before and after biological loading) and between the groups (CTRL, DENT, PRIM, BOND, PEAK). Factors “adhesive treatment” (p = 0.001) and “biological loading” (p = 0.005) significantly influenced microtensile bond strength. DENT, BOND (p = 0.001), and PEAK (p = 0.008) μTBS were significantly higher at baseline than after biological loading.

**Fig 4:**
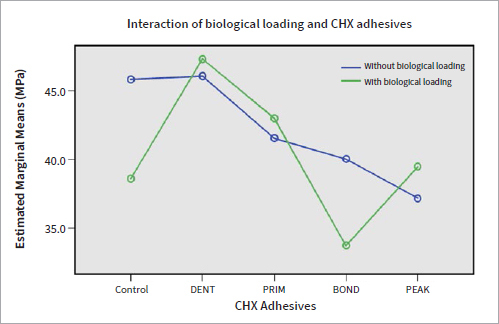
Correlation of biological loading in the caries model and type of CHX adhesive. Bond strengths for DENT and PRIM are similar with or without biological loading.

**Table 3:** Mean μTBS at baseline and after biological loading in the caries model

Groups	Baseline		Caries model	
	No. of sticks	MPa [SD]	No. of sticks	MPa [SD]
CTRL	99	58.8 [19.6]^A,a^	102	30.4 [16.9]^B,b^
DENT	105	54.0 [18.2]^Aa,b^	109	45.6 [17.7]^Ba^
PRIM	108	45.7 [16.1]^Ac^	99	40.1 [13.4]^Aa^
BOND	106	49.6 [18.6]Ab	107	32.4 [13.1]^Bb^
PEAK	103	45.2 [15.4]^Ac^	105	40.8 [14.9]^Ba^

Means followed by the same capital letters in rows (comparison within groups) and the same lowercase letters in columns (comparison between groups) do not differ statistically significantly (p > 0.05, Bonferroni).

### Baseline

**Fig 5:**
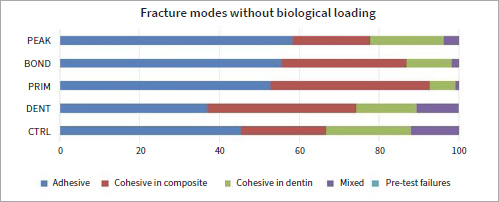
Modes of bond failure without biological loading (baseline). Different fractures modes are presented in %. Most failures for all groups are adhesive.

Regarding comparisons between the groups, CTRL exhibited significantly higher μTBS compared to PRIM (p = 0.000), BOND (p = 0.002), and PEAK (p = 0.000), while DENT exhibited better performance than PRIM (p = 0.006) and PEAK (p = 0.004) (Table 3). Moreover, DENT showed a significantly lower percentage of adhesive fractures compared to PRIM (p = 0.000) and PEAK (p = 0.000). No difference between fracture modes was evident between CTRL and the other experimental groups (p > 0.05) (Fig 5).

### Artificial Mouth/Caries Model

**Fig 6:**
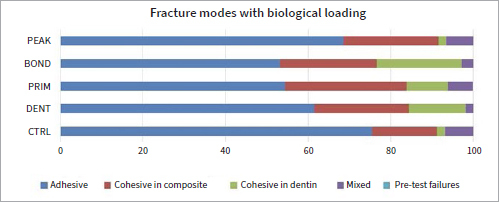
Modes of bond failure with biological loading. Different fractures modes are presented in %. Adhesive fractures increased compared to baseline.

CTRL and BOND showed the lowest µTBS after biological loading in the caries model (p < 0.05). CTRL demonstrated significantly lower μTBS compared to DENT (p = 0.000), PRIM (p = 0.008) and PEAK (p = 0.000). Lower values were also found for BOND in comparison to DENT (p = 0.000), PRIM (p = 0.003), and PEAK (p = 0.001) (Table 3). DENT (p = 0.041), PRIM (p = 0.000), and BOND (p = 0.000) exhibited a lower percentage of adhesive and a higher percentage of cohesive fractures than CTRL, while PEAK showed a significantly higher percentage of adhesive fractures than did PRIM (p = 0.000) and BOND (p = 0.027) (Fig 6).

No interaction was found between bond strength values and fracture modes (p = 0.358, Pearson correlation). After biological loading, DENT (p = 0.041), PRIM (p = 0.000), and BOND (p = 0.000) exhibited significantly lower percentages of less adhesive fractures than CTRL.

**Fig 7:**
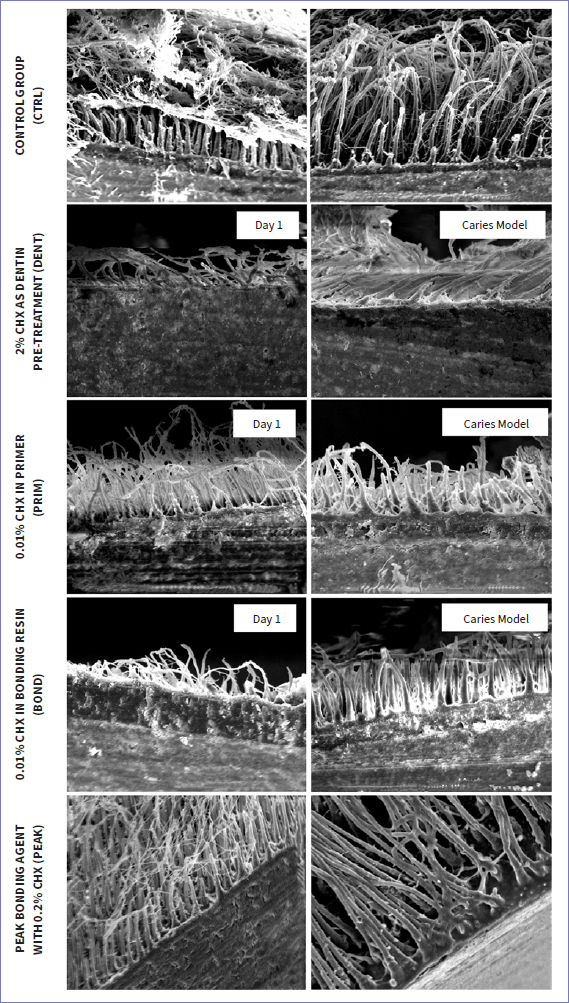
SEM images of the adhesive interfaces at 1000X magnification at baseline (left) and after biological loading in the caries model (right) for tested groups.

Qualitative SEM evaluation of representative specimens was performed at 1000X magnification. Compared to baseline, no alteration in presence of resin tags, width of hybrid layer, or quality of the adhesive interface was evident after biological loading in the caries model, compared to baseline (Fig 7).

## Discussion

Considering that adding CHX to restorative materials is related to side-effects regarding their physicomechanical properties,^15,16,26,43^ the purpose of the present study was to test CHX addition in adhesives, rather than in restorative materials, and its potential antibacterial action by means of a bacterial caries model. CHX was used as dentin pretreatment (DENT), added to the primer (PRIM) or the bonding agent (BOND), and tested in a commercially available universal adhesive (PEAK). CTRL (=0.009), DENT (p = 0.001), BOND (p = 0.001), and PEAK (p = 0.008) showed better adhesion at baseline compared to their μTBS after biological loading. Comparisons between the groups at baseline showed lower bond strengths for PRIM (p = 0000), BOND (p = 0.002), and PEAK (p = 0.000) compared to CTRL, as well as for PRIM (p = 0.006) and PEAK (0.004) compared to DENT. After biological loading, DENT, PRIM, and PEAK showed higher μTBSs than both CTRL and DENT (p < 0.05). Therefore, the null hypothesis was rejected.

It has been hypothesized that CHX adhesives can not only minimize secondary caries progression due to its antibacterial action upon release from the adhesives, they also inhibit adhesive bond degradation due to their anti-proteolytic effect. For the first hypothesis, it is mandatory that CHX be released from the adhesive interface and act extrinsically at the resin-dentin interface, since bacteria gather at the surface of the restoration or restoration margins. On the other hand, the second hypothesis requires that CHX remain within dentin, exerting its protective activity against collagenolytic enzymes, which are activated by caries.^52^ However, since both effects take place simultaneously in the same experimental set-up, and little information exists on the kinetics of CHX,^14,24^ it is impossible to distinguish the extent to which the intrinsic (anti-proteolytic) or the extrinsic (antibacterial) action of CHX is responsible for the µTBS results after biological loading in the caries model. Since the amount of added CHX is limited to protect the physicomechanical properties of the adhesives, it is logical that these two scenarios are antagonistic. CHX diglugonate was admixed to the primer and bonding agent of a commercially available 3-step adhesive, according to the methodology of previous studies.^55,56^ Some authors^55,56^ hypothesized that adhesives containing CHX could carry the latter deeper into the hybrid layer, offering slower CHX release due to its depth (and thus longer antimicrobial action), as well as bring it closer to the source of endogenous proteases (MMPs and CCs), which would increase its anti-collagenolytic action. Since the adhesive inteface is the weakest link of composite restorations, loading adhesives instead of restorative materials with antimicrobials offers a localized action in the weakest area. Since the literature presents controversial results regarding degree of conversion,^11,37^ elasticity,^11^ water sorption,^18,51^ and bond strength^18,44,50,54,56^ when CHX is admixed into adhesives in concentrations up to 5%, a safe concentration of 0.1% CHX was chosen for the present study for the experimental adhesives as one of the lowest evaluated in literature, which can potentially show positive effects. According to the baseline results of the present study, experimentally adding CHX to the primer or bonding agent, or when already industrially added in a universal adhesive, failed to produce higher or even similar bond strengths compared to the control group (p < 0.01), suggesting a potential interference with the mechanical properties of the adhesives. On the other hand, it is unclear whether this low – but safe – CHX concentration would be able to induce an antibacterial and anti-proteolytic effect. According to Gendron et al,^21^ only a much lower CHX concentration of 0.0001% is necessary to suppress MMP-2 activity, 0.002% for MMP-9 and 0.02% for MMP-8. The universal adhesive PEAK was the only adhesive of the study that contained 0.2% CHX, compared to the experimental adhesives PRIM and BOND which contained 0.1%. These values mentioned in the literature,^21^ however, correspond to the adequate CHX concentration at the site of action and not to the CHX concentration initially delivered on dentin or added to the adhesives.

Moreover, since CHX concentration changes over time due to kinetics, the duration of on-site CHX delivery is questionable, even in the above-mentioned minimal amounts, regardless of its initial concentration. According to one study, CHX release was detectable for up to 5 weeks,^24^ and a different study showed some decrease in bacterial counts up to 3 months.^14^ Both observation times cover the timeframe of the present experiment (1 day of storage and 2 days of loading) testing CHX’s antibacterial and antiproteolytic action. Due to our short observation time, CHX adhesives were expected to show favorable results in terms of protection of bond strength. This is a limitation of the present study, as long-term monitoring of CHX adhesives was not included.

A mono-bacterial, automated caries model established in earlier studies^1,6,31,49^ was used, loading teeth with 8 demineralization phases within 2 days. In order to produce sufficient demineralization, the pH of the bacterial solution should be 4.2 to 4.3.^1,6,31,49^ This enables sufficient demineralization of both enamel and dentin. Each demineralization lasted for 1 h, and specimens were incubated with *S. mutans* for 4 h each day. The effect of intraoral sugar clearance was simulated by rinsing the reaction chamber three times with artificial saliva after each demineralization, removing bacterial remnants which would otherwise impede a rise in pH.

For μTBS testing, the number of specimens (99–109 sticks/group) was larger than in similar studies with CHX adhesives (10-40 sticks/group).^44,50,54-57^ The classification of failure modes after μTBS testing is related to the level of magnification employed, as a failure that is considered adhesive under low magnification (as with the Magnifier Lamp mentioned above) can be identified as cohesive or mixed when evaluated under an optical microscope, where small composite or dentin remnants on the adhesive interface would be visible. While this may result in fewer adhesive failures, there is no standardization of the level of magnification for μTBS studies. In the present study, the level of magnification (4X) was in accordance with relevant literature.^54-57^

Adhesives containing CHX (PRIM, BOND, PEAK) exhibited lower bond strengths at baseline compared to control (p < 0.01), showing a potentially negative interaction between the adhesive components of experimental adhesives and CHX. Regarding PEAK, despite the fact that CHX was industrially added to the adhesive, its distinct composition as a universal adhesive compared to the etch-and-rinse approach of Scotchbond Multipurpose (which was used in the other groups and lacks functional monomers), may have also played a role in its lower bond strengths. When 2% CHX was applied as dentin pre-treatment, no such decrease in bond strength was observed (p > 0.05) (Table 3, Fig 3). Despite the fact that CHX may reduce the number of *S. mutans* when applied as dentin pre-treatment,^28^ this study showed that CHX adhesives were not able to protect bond strength from deterioration after biological loading; μTBS did not differ significantly for CTRL and PRIM (p > 0.05) before and after biological loading (comparison within the groups), and lower µTBS was found after biological loading for DENT, BOND, and PEAK (p < 0.01) compared to their respective baseline values (Table 3). The decreased bond strength after biological loading for DENT, BOND, and PEAK correlated well with the existing literature, which has shown that cariogenic bacteria can degrade dental resin composites and adhesives^7^ and negatively affect μTBS.^35^ It may be possible that CHX adhesives failed to counteract the bacterial action of *S. mutans* either because the CHX concentration was not sufficient or it could not be released from the adhesives and delivered adquately. However, qualitative evaluation of the hybrid layer of representative specimens of each CHX group did not show any alteration in the appearance of the hybrid layer or presence of resin tags after loading in the caries model compared to the baseline (Fig 7). In contrast, Borges et al^4^ found no reduction of adhesive bond strengths after a 4 h/day cariogenic challenge. However, those authors did not take into account alternating demineralization and remineralization phases, and although the duration of demineralization was the same as in the present study, the cariogenic challenge did not simulate oral cavity conditions. The bond strength of CHX adhesives after cariogenic challenge varied between the groups. DENT, PRIM, and PEAK exhibited significantly better performance compared to the control group (CTRL) (p < 0.01), indicating a protective effect of CHX against bacterial degradation of the adhesive bond (comparison between the groups). DENT also exhibited fewer adhesive fractures in both situations, at baseline and after biological loading) (p < 0.05) (Fig 5, 6), confirming the fact that no negative impact existed in the adhesive interface. Due to a lack of similar studies, further research is needed regarding biological loading of CHX adhesives. Research should be focussed on testing higher CHX concentrations in the adhesives, in a form of delivery that would provide controlled release and would not harm the material’s properties.

## Conclusion

CHX adhesives showed lower bond strengths after biological loading compared to their baseline μTBS, showing that 2% CHX solution used as dentin pre-treatment, and addition of CHX to a bonding agent or in a universal adhesive failed to protect against bacteria and collagenolytic enzymes. However, addition of CHX to the primer showed no significant deterioration after the caries model, possibly suggesting some clinical advantage.

The baseline μTBS of adhesives with admixed CHX (PRIM, BOND, and PEAK) was lower than the control, suggesting a potential interference of CHX with the other adhesive components. Therefore, 2% CHX as a dentin pre-treatment seems to be a safer option.

After biological loading, 2% CHX as dentin pretreatment and addition of CHX to the primer or in a universal adhesive showed higher μTBS compared to the control, suggesting a possible protective effect of CHX against bond degradation due to bacteria and collagenolytic enzymes.

Addition of CHX to the bonding agent failed to protect the adhesive zone and is possibly related to bond strength deterioration due to interference with the adhesive’s mechanical properties.
